# Development and validation of an ultrasound-based deep learning radiomics nomogram for predicting the malignant risk of ovarian tumours

**DOI:** 10.1186/s12938-024-01234-y

**Published:** 2024-04-09

**Authors:** Yangchun Du, Yanju Xiao, Wenwen Guo, Jinxiu Yao, Tongliu Lan, Sijin Li, Huoyue Wen, Wenying Zhu, Guangling He, Hongyu Zheng, Haining Chen

**Affiliations:** 1 Department of Ultrasound, The People’s Hospital of Guangxi Zhuang Autonomous Region and Guangxi Academy of Medical Sciences, No. 6 Taoyuan Road, Qingxiu District, Nanning, 530021 China; 2 Department of Pathology, The People’s Hospital of Guangxi Zhuang Autonomous Region and Guangxi Academy of Medical Sciences, No. 6 Taoyuan Road, Qingxiu District, Nanning, 530021 China

**Keywords:** Ultrasound, Ovarian tumour, O-RADS, DLR_Nomogram

## Abstract

**Background:**

The timely identification and management of ovarian cancer are critical determinants of patient prognosis. In this study, we developed and validated a deep learning radiomics nomogram (DLR_Nomogram) based on ultrasound (US) imaging to accurately predict the malignant risk of ovarian tumours and compared the diagnostic performance of the DLR_Nomogram to that of the ovarian-adnexal reporting and data system (O-RADS).

**Methods:**

This study encompasses two research tasks. Patients were randomly divided into training and testing sets in an 8:2 ratio for both tasks. In task 1, we assessed the malignancy risk of 849 patients with ovarian tumours. In task 2, we evaluated the malignancy risk of 391 patients with O-RADS 4 and O-RADS 5 ovarian neoplasms. Three models were developed and validated to predict the risk of malignancy in ovarian tumours. The predicted outcomes of the models for each sample were merged to form a new feature set that was utilised as an input for the logistic regression (LR) model for constructing a combined model, visualised as the DLR_Nomogram. Then, the diagnostic performance of these models was evaluated by the receiver operating characteristic curve (ROC).

**Results:**

The DLR_Nomogram demonstrated superior predictive performance in predicting the malignant risk of ovarian tumours, as evidenced by area under the ROC curve (AUC) values of 0.985 and 0.928 for the training and testing sets of task 1, respectively. The AUC value of its testing set was lower than that of the O-RADS; however, the difference was not statistically significant. The DLR_Nomogram exhibited the highest AUC values of 0.955 and 0.869 in the training and testing sets of task 2, respectively. The DLR_Nomogram showed satisfactory fitting performance for both tasks in Hosmer–Lemeshow testing. Decision curve analysis demonstrated that the DLR_Nomogram yielded greater net clinical benefits for predicting malignant ovarian tumours within a specific range of threshold values.

**Conclusions:**

The US-based DLR_Nomogram has shown the capability to accurately predict the malignant risk of ovarian tumours, exhibiting a predictive efficacy comparable to that of O-RADS.

**Supplementary Information:**

The online version contains supplementary material available at 10.1186/s12938-024-01234-y.

## Background

Ovarian tumours are common diseases of the female reproductive system and can be categorised into benign and malignant types according to their characteristics [1]. Benign ovarian tumours exhibit an extended disease trajectory and gradual growth rate, enabling the exploration of conservative treatment options. Conservative treatment circumvents superfluous expenditures and aggressive interventions while safeguarding the reproductive potential of young patients. Conversely, the management of malignant ovarian tumours requires the expertise of a gynaecologic oncologist, entailing specialised diagnostic processes and treatment regimens that include accurate staging and radical surgical procedures [2]. The timely identification and management of ovarian cancer are critical determinants of patient prognosis. The 5-year overall survival rate for ovarian cancer is 46%, with a significant disparity between late-stage (29%) and early-stage (92%) diagnoses [3]. Consequently, an accurate distinction between benign and malignant ovarian tumours is paramount for devising tailored and efficacious treatment strategies [4].

The detection and management of ovarian cancer rely heavily on blood serum tumour markers, with carbohydrate antigen 125 (CA125) widely acknowledged as a crucial biomarker for monitoring epithelial ovarian cancer. However, its sensitivity and specificity are relatively low. CA125 levels may increase due to various physiological or pathological factors, including menstruation, pregnancy, endometriosis, and peritoneal inflammatory diseases [5]. Inflammation is widely acknowledged as a prominent feature of cancer advancement and evolution [6]. Numerous risk factors linked to the aetiology of ovarian cancer are directly or indirectly associated with inflammation, indicating the potential involvement of inflammation in ovarian cancer development [3, 7–9]. The prognostic factors associated with inflammation, including the platelet-to-lymphocyte ratio (PLR), neutrophil-to-lymphocyte ratio (NLR), derived neutrophil-to-lymphocyte ratio (dNLR), lymphocyte-to-monocyte ratio (LMR), systemic immune-inflammation index (SII), and C-reactive protein–albumin ratio (CAR), have been assessed for their prognostic value in diverse cohorts of patients with solid cancers [10–13]. Studies have demonstrated the significance of these factors in predicting the malignancy potential, staging, and prognosis of ovarian tumours [14, 15].

Ultrasound (US) is the primary imaging technique for enhancing the precision of ovarian or adnexal mass diagnoses [1]. However, the diagnosis accuracy of US is contingent on the subjective interpretation and expertise of US experts. Regrettably, proficient US specialists are scarce, resulting in inconsistent diagnostic accuracy among technicians with different experience levels [16]. The American College of Radiology (ACR) has formally published the consensus guidelines for ovarian-adnexal reporting and data system (O-RADS) US risk stratification and management to establish a standardised terminology for describing the characteristics of ovarian-adnexal masses in US reports and enhance the accuracy of assessing the malignant risk associated with such tumours [17]. Cao et al. [18] conducted a study to validate the O-RADS US risk stratification consensus by analysing 1054 adnexal masses. Their findings suggested that this consensus is a valuable tool for effectively stratifying the malignancy risk of adnexal tumours, even among experts with varying experience levels. The study also revealed that the O-RADS categories 4 and 5 had the highest proportions of malignant adnexal tumours, accounting for 34.46% and 89.57%, respectively. The O-RADS classification’s area under the ROC curve (AUC) in predicting malignant adnexal tumours was 0.960, with an optimal cut-off value greater than O-RADS 3. However, a significant number of patients presented with benign lesions in the O-RADS 4 category. Therefore, it is crucial to accurately identify the malignancy risk of ovarian tumours classified as O-RADS 4 and O-RADS 5 categories to deliver precise diagnoses and treatment alternatives.

Recently, radiomics has emerged as an evolving and crucial domain in the analysis of medical images, offering a novel approach for transforming these images into quantitative characteristics that reveal tumour-related biological information. These characteristics can be scrutinised to enhance clinical decision-making [19]. Manual radiomic methods extract only clearly defined and surface-level image features that inadequately capture the heterogeneity of tumours, thereby constraining the potential of radiomics [20]. Deep learning (DL) has recently attracted considerable interest in the medical field. DL is a technology encompassing multiple layers rather than being a singular algorithm. The convolutional neural network (CNN) is the most frequently employed DL network in medical image research and exhibits remarkable efficacy in image segmentation and classification [21]. Previous studies have indicated that CNNs can provide diverse high-level semantic features of images relevant to specific clinical outcomes [22]. However, the effective implementation of DL requires a substantial amount of training data, which is often lacking in medical datasets owing to their limited size or scale. Consequently, many practical applications currently employ pre-trained CNN, a transfer learning (TL) technique, as an alternative to DL to mitigate the issue of overfitting caused by inadequate training data [23, 24]. Integrating deep transfer learning (DTL) classification networks with conventional manual radiomics frameworks has gained popularity in medical research [25, 26]. However, limited research exists on applying this approach to ovarian tumours, primarily on computed tomography imaging [27].

This study aimed to develop and validate a US-based combined model, the deep learning radiomics nomogram (DLR_Nomogram), as a decision-support tool for the preoperative discrimination of the malignant risk of ovarian tumours. Furthermore, the predictive performance of the DLR_Nomogram was compared with that of the O-RADS.

## Results

### The clinical baseline data

Eight hundred forty-nine patients diagnosed with ovarian tumours were enrolled for task 1. The study cohort was randomly divided into training and testing sets at a ratio of 8:2. The distribution of patients with benign and malignant ovarian tumours remained relatively consistent across the entire study population and within the training and testing sets. The baseline characteristics of task 1 are shown in Additional file 1: Table S1.

### The O-RADS predicted the malignant risk of ovarian tumours

The AUC of the O-RADS classification for predicting the malignancy risk of ovarian tumours was 0.960 (95% CI: 0.947–0.972, *P* < 0.05), with the optimal cut-off value being > O-RADS 3. Based on the statistical analysis, O-RADS 4 and 5 lesions showed malignancy, with an accuracy, sensitivity, and specificity of 88.1%, 98.3%, and 82.5%, respectively, as depicted in Additional file 2: Fig. S1.

As shown in Fig. 7, benign tumours accounted for a larger percentage (48.65%) of ovarian-adnexal lesions categorised as O-RADS 4. To tackle this problem, we devised task 2, which entailed developing and validating a DLR-Nomogram for predicting the probability of malignancy in O-RADS 4 and O-RADS 5 ovarian lesions. Out of 391 people studied, 96 (24.55%) had benign ovarian tumours, and 295 (75.45%) had malignant ovarian tumours. The study population was randomly divided into training and testing sets in an 8:2 ratio. The baseline characteristics of the participants are provided in Additional file 1: Table S2.

### Construction of clinical signature

Additional file 1: Tables S1 and S2 display the clinical parameters and US semantic features that exhibited significant differences between benign and malignant ovarian tumours in tasks 1 and 2 of the training set. No evident linear relationship was observed among these parameters through spearman correlation analysis (Additional file 3: Fig. S2). These parameters were selected to create the clinical signature (Clinic_Sig).

### The extraction and selection of manual radiomics features and construction of radiomics signature

A total of 1476 handcrafted radiomics features were extracted from tasks 1 and 2, including the first-order features, shape features, gray-level dependence matrix (GLDM), gray-level size zone matrix (GLSZM), gray-level run length matrix (GLRLM), and gray-level co-occurrence matrix (GLCM). The number and proportion of handcrafted radiomics features are presented in Additional file 4: Fig. S3. The *P*-value results for all features are shown in Additional file 5: Fig. S4.

**Fig. 1:**
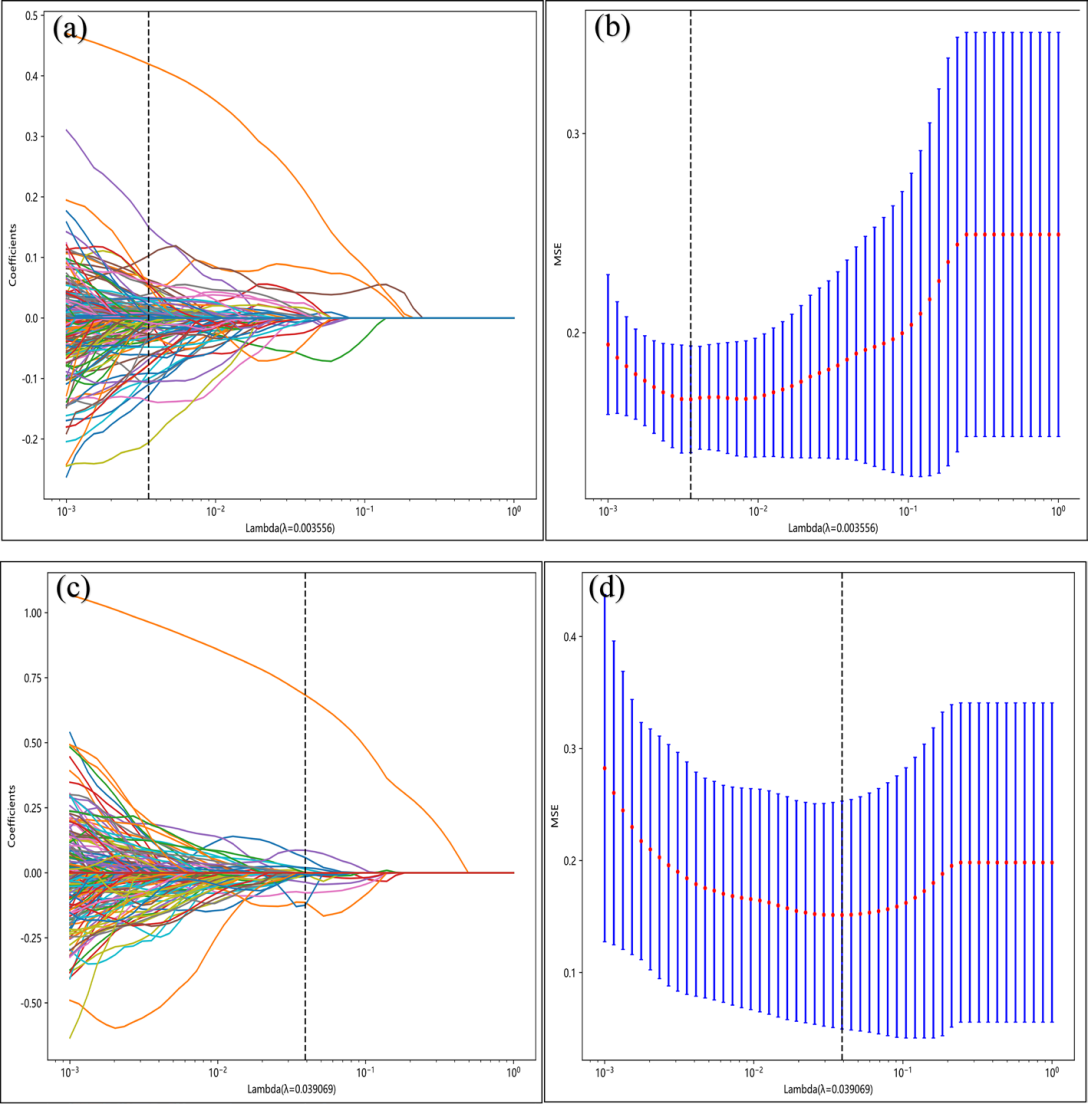
Coefficients and MSE of tenfold cross-validation. **a** and **b**: task 1; **c** and **d**: task 2. Note: MSE, mean square error

An initial screening of features with an intra-/inter-class correlation coefficient (ICC) ≥ 0.85 and retained features with *P* < 0.05 was conducted using t-tests or Mann–Whitney U tests, and 1161 and 1105 features were included for tasks 1 and 2, respectively. Subsequently, spearman correlation analysis and the greedy recursive feature removal strategy were conducted, and 301 and 254 features were retained for tasks 1 and 2, respectively. Next, the least absolute shrinkage and selection operator (LASSO) regression was applied with a tenfold cross-validation using the minimum criterion to identify the optimal *λ* values of 0.003556 and 0.039069 for tasks 1 and 2, respectively. These *λ* values yielded the minimum cross-validation errors, as shown in Fig. 1. Subsequently, 99 and 16 nonzero coefficient features were employed for tasks 1 and 2, respectively (Additional file 6: Fig. S5). Finally, the best features were input into the LR model, and the radiomics signature (Rad_Sig) was constructed using fivefold cross-validation.

### Construction of deep transfer learning radiomic signature

The two-dimensional US images of the maximum section of ovarian tumours were cropped and input into a pre-trained CNN. The hidden layer algorithm of the CNN was applied, and the resulting predicted probability of malignancy risk for each ovarian tumour was labelled deep transfer learning radiomic signature (DTL_Sig). A testing set was used to determine the optimal training outcomes. Ultimately, the resnet50 and densenet121 models were selected to predict the malignancy risk of ovarian tumours in tasks 1 and 2, respectively.

**Fig. 2:**
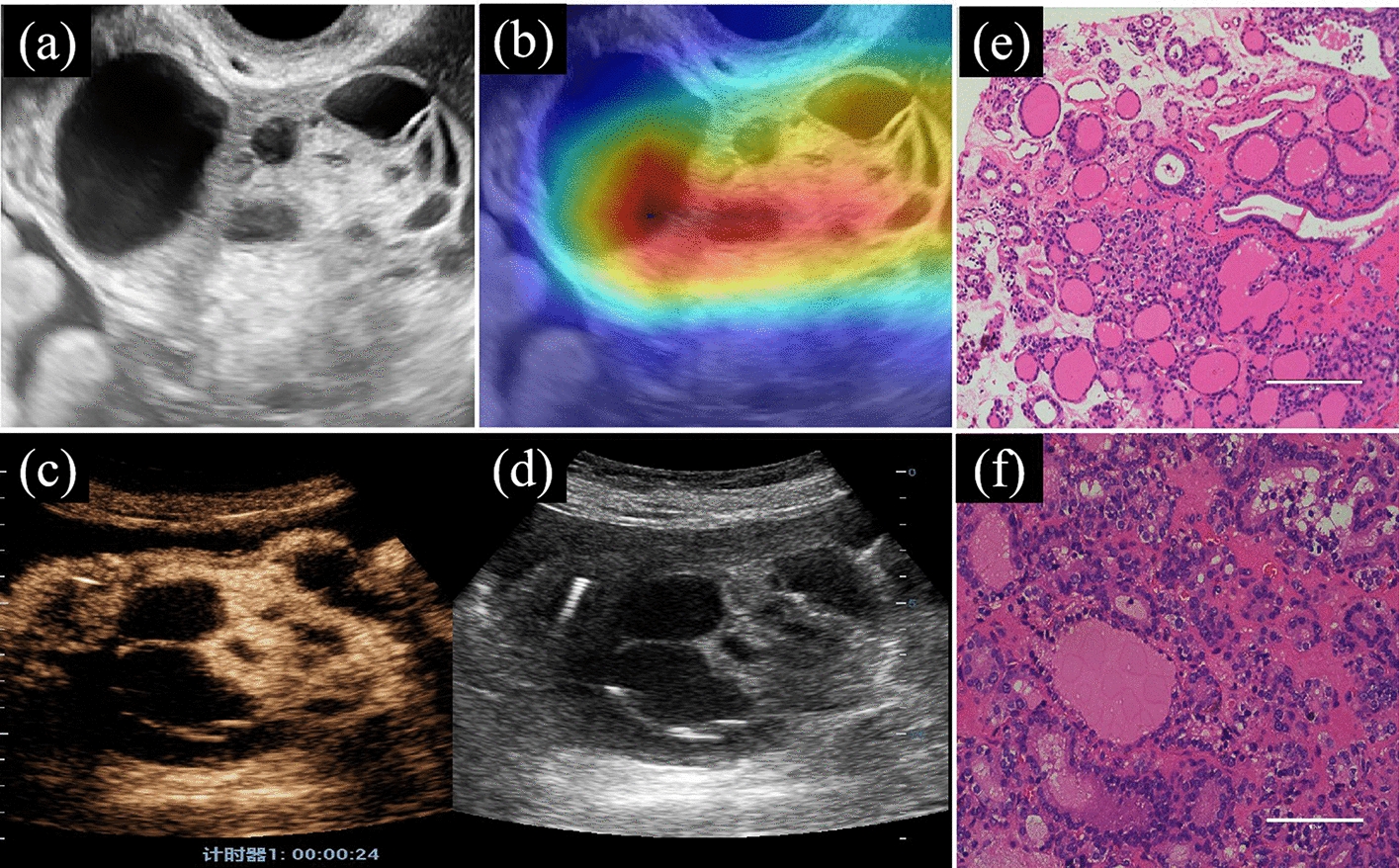
The CNN model with Grad-CAM was used on the ovarian tumour. **a** and **d**: Two-dimensional US image; **b**: Grad-CAM, the red area displayed the basis of decision-making of CNN; **c**: CEUS; **e** and **f**: histopathological results: struma ovarii (20× and 40×, respectively). Note: Grad-CAM, gradient-weighted class activation mapping; CNN, convolutional neural network; CEUS, contrast-enhanced ultrasound

However, the DTL algorithm is commonly regarded as a “black box” owing to its opaque internal processes. Gradient-weighted class activation mapping (Grad-CAM) was employed to visually represent the network’s inner workings and elucidate the CNN algorithm’s decision-making process. It involved generating a rudimentary localisation map highlighting the pivotal focus areas for CNN’s decision to predict the malignancy probability of ovarian tumours, denoted by red regions. It is accomplished by extracting the feature layer at the end of the network model and performing a weighted sum of all feature maps to obtain a final heatmap [28]. The last convolutional layer of the final res-block was made transparent to predict the malignancy risk of ovarian tumours, as shown in Fig. 2.

The images in Fig. 2 demonstrate a cystic and solid mixed echoic mass measuring 100 mm in diameter in one patient’s pelvis. The CA125 level was 601 U/mL. Contrast-enhanced ultrasound (CEUS) revealed the contrast agent perfusion into the solid portion of the lesion, with early and high enhancement observed in the ovarian lesions compared with the adjacent myometrium at the same level. The US expert considered that it was a malignant ovarian tumour (O-RADS 5). Nevertheless, there was a 91.8% probability that the lesions were benign, according to the DTL_Sig analysis. The histopathological findings confirmed a benign ovarian tumour, struma ovarii with ossification. Remarkably, the predicted probability obtained from the DTL_Sig analysis strongly aligned with the histopathological diagnosis.

### Construction of the DLR_Nomogram

**Fig. 3:**
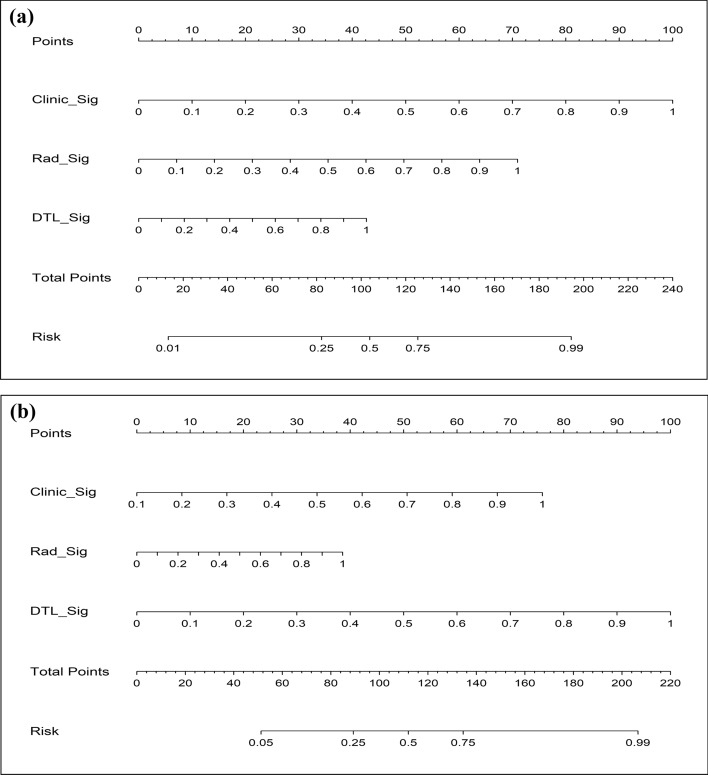
The DLR_Nomogram integrating the prediction results of Clinic_Sig, Rad_Sig and DTL_Sig. **a** task 1; **b** task 2. Note: DLR, deep learning radiomics; Clinic_Sig, clinical signature; Rad_Sig, radiomics signature; DTL_Sig, deep transfer learning radiomic signature

The predicted probabilities of Rad_Sig, DTL_Sig, and Clinic_Sig for each sample were aggregated and used as input variables for the LR model to develop a composite model for the training dataset. Subsequently, the combined model was graphically represented as the DLR_Nomogram (Fig. 3), with the primary objective of discerning the malignancy risk of ovarian tumours.

### Model evaluation

**Fig. 4:**
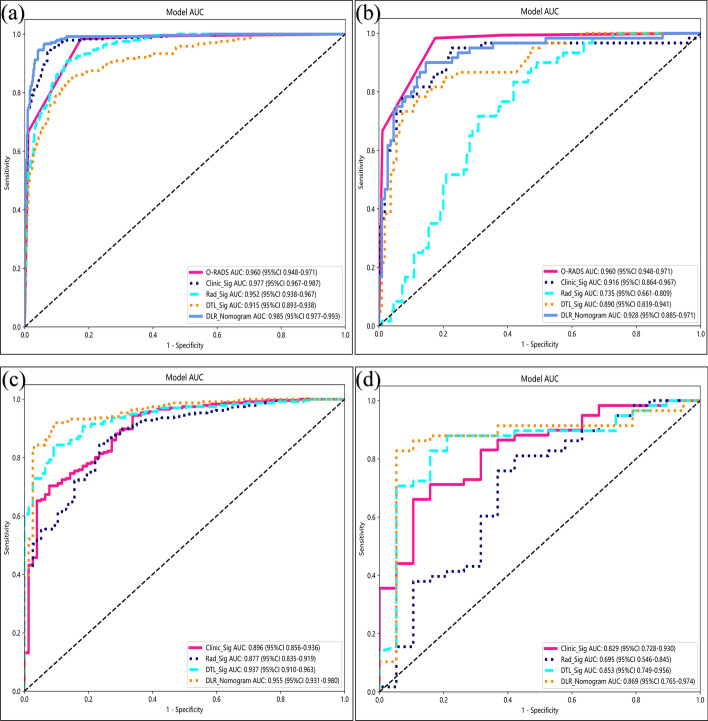
The ROC of the O-RADS, Clinic_Sig, Rad_Sig, DTL_Sig and DLR_Nomogram. **a** and **b**: The training and testing sets of task 1, respectively; **c** and **d**: the training and testing sets of task 2, respectively. Note: ROC, receiver operating characteristic curve; Clinic_Sig, clinical signature; Rad_Sig, radiomics signature; DTL_Sig, deep transfer learning radiomic signature; DLR_Nomogram, deep learning radiomic nomogram

The AUC, accuracy, precision, recall, and F1 score of O-RADS, Clinic_Sig, Rad_Sig, DTL_Sig, and DLR_Nomogram for the prediction malignancy risk of ovarian tumours are presented in Table 1, with the corresponding receiver operating characteristic curve (ROC) shown in Fig. 4. In task 1, the AUC value of O-RADS was higher than that of the DLR_Nomogram in the testing set, however, the Delong test (Table 2) did not show a statistically significant difference between the two groups (*P* > 0.05). In task 2, the AUC values of the DLR_Nomogram were the highest both for the training and testing sets.

**Table 1:** The performance of models predicting the malignancy risk of ovarian tumours

	O-RADS	Clinic_Sig	Rad_Sig	DTL_Sig	DLR_Nomogram
Task 1					
Training set					
AUC	0.960	0.977	0.952	0.915	0.985
2.5%CI	0.948	0.967	0.938	0.893	0.977
97.5%CI	0.971	0.987	0.967	0.938	0.993
Accuracy	0.881	0.925	0.884	0.860	0.948
Precision	0.754	0.895	0.843	0.819	0.925
Recall	0.983	0.892	0.825	0.775	0.929
F1 score	0.854	0.894	0.834	0.797	0.927
Testing set					
AUC	0.960	0.916	0.735	0.890	0.928
2.5%CI	0.948	0.864	0.661	0.839	0.885
97.5%CI	0.971	0.967	0.809	0.941	0.971
Accuracy	0.881	0.859	0.671	0.841	0.871
Precision	0.754	0.875	0.530	0.800	0.880
Recall	0.983	0.700	0.583	0.733	0.733
F1 score	0.854	0.778	0.556	0.765	0.800
Task 2					
Training set					
AUC		0.896	0.877	0.937	0.955
2.5%CI		0.857	0.835	0.911	0.931
97.5%CI		0.936	0.919	0.963	0.980
Accuracy		0.872	0.789	0.879	0.888
Precision		0.889	0.917	0.896	0.900
Recall		0.949	0.792	0.949	0.958
F1 score		0.918	0.850	0.922	0.928
Testing set					
AUC		0.829	0.695	0.853	0.869
2.5%CI		0.728	0.546	0.749	0.765
97.5%CI		0.930	0.845	0.956	0.974
Accuracy		0.808	0.714	0.844	0.779
Precision		0.867	0.846	0.926	0.815
Recall		0.881	0.759	0.862	0.914
F1 score		0.874	0.800	0.893	0.862

Abbreviations: *O-RADS* ovarian-adnexal reporting and data system, *CI* confidence interval, *Clinic_Sig* clinical signature, *Rad_Sig* radiomics signature, *DTL_Sig* deep transfer learning radiomic signature, *DLR_Nomogram* deep learning radiomic nomogram, *AUC* area under the curve of ROC

**Table 2:** The DeLong test of models

	Task 1		Task 2	
	Training set	Testing set	Training set	Testing set
O-RADS *vs* Clinic_Sig	*P* < 0.001	*P* > 0.05		
O-RADS *vs* Rad_Sig	*P* > 0.05	*P* < 0.001		
O-RADS *vs* DTL_Sig	*P* < 0.001	*P* < 0.05		
O-RADS *vs* DLR_Nomogram	*P* < 0.001	*P* > 0.05		
Clinic_Sig *vs* Rad_Sig	*P* < 0.05	*P* < 0.001	*P* > 0.05	*P* > 0.05
Clinic_Sig *vs* DTL_Sig	*P* < 0.001	*P* > 0.05	*P* > 0.05	*P* > 0.05
Clinic_Sig *vs* DLR_Nomogram	*P* < 0.05	*P* > 0.05	*P* < 0.001	*P* > 0.05
Rad_Sig *vs* DTL_Sig	*P* < 0.001	*P* < 0.001	*P* < 0.05	*P* < 0.05
Rad_Sig *vs* DLR_Nomogram	*P* < 0.001	*P* < 0.001	*P* < 0.001	*P* < 0.05
DTL_Sig *vs* DLR_Nomogram	*P* < 0.001	*P* > 0.05	*P* > 0.05	*P* > 0.05

Abbreviations: *O-RADS* ovarian-adnexal reporting and data system, *Clinic_Sig* clinical signature, *Rad_Sig* radiomics signature, *DTL_Sig* deep transfer learning radiomic signature, *DLR_Nomogram* deep learning radiomic nomogram; *vs* versus

**Fig. 5:**
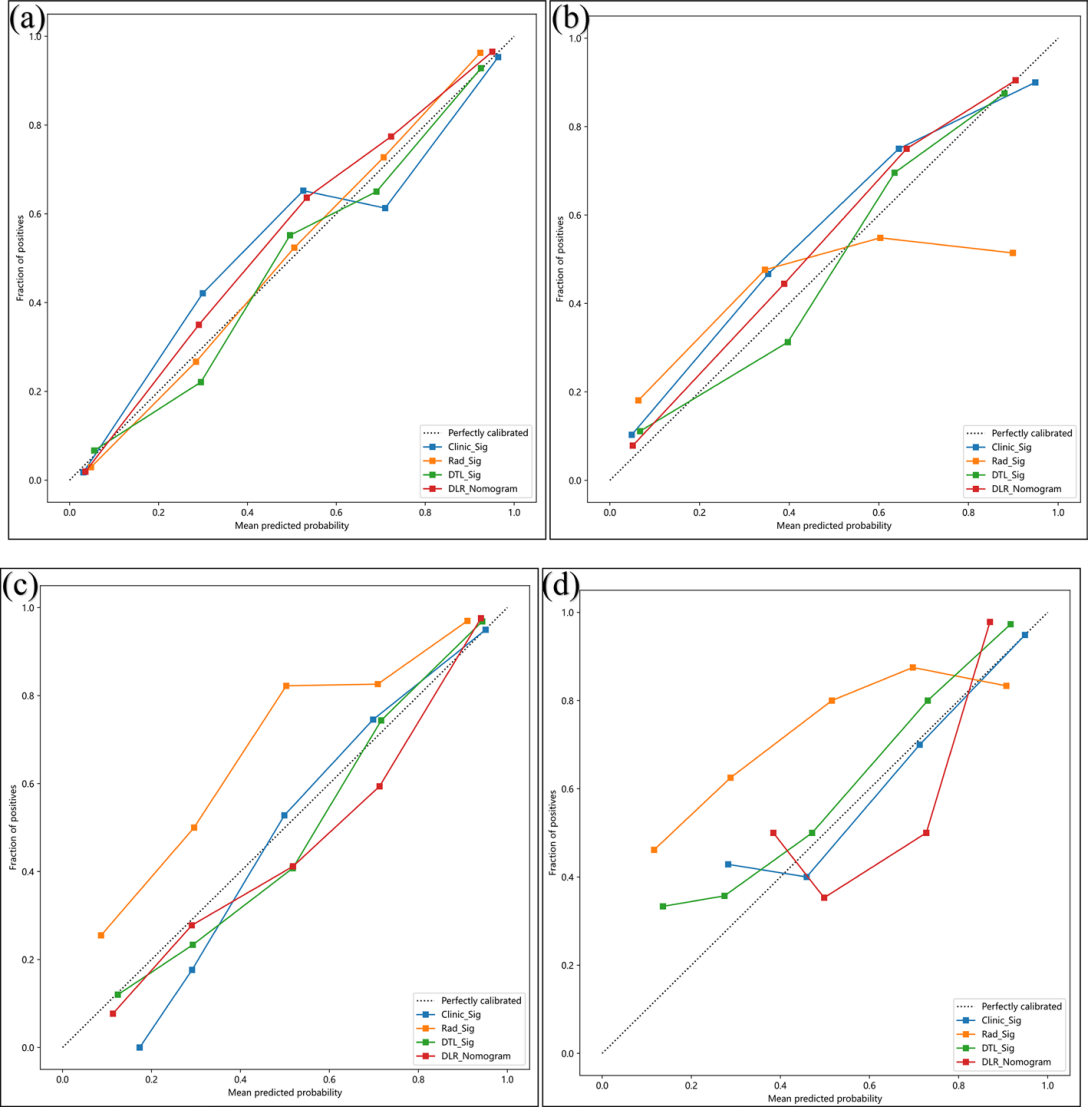
Calibration curves of the four models. **a** and **b**: The training and testing sets of task 1, respectively; **c** and **d**: the training and testing sets of task 2, respectively. Note: Clinic_Sig, clinical signature; Rad_Sig, radiomics signature; DTL_Sig, deep transfer learning radiomic signature; DLR_Nomogram, deep learning radiomic nomogram

The Hosmer–Lemeshow (HL) test was used to evaluate the degree of concordance between the projected and observed values of the model, as shown in Table 3. The *P*-values of the DLR_Nomogram in the training and testing sets > 0.05 in tasks 1 and 2, indicating that the DLR_Nomogram exhibited a strong level of concordance in predicting the malignancy risk of ovarian tumours, as evidenced by the calibration curves depicted in Fig. 5.

**Table 3:** The HL test of models

	Task 1		Task 2	
	Training set	Testing set	Training set	Testing set
Clinic_Sig	*P* > 0.05	*P* < 0.001	*P* < 0.001	*P* > 0.05
Rad_Sig	*P* > 0.05	*P* < 0.001	*P* < 0.001	*P* < 0.001
DTL_Sig	*P* < 0.05	*P* > 0.05	*P* > 0.05	*P* > 0.05
DLR_Nomogram	*P* > 0.05	*P* > 0.05	*P* > 0.05	*P* > 0.05

Abbreviations: *Clinic_Sig* clinical signature, *Rad_Sig* radiomics signature, *DTL_Sig* deep transfer learning radiomic signature, *DLR_Nomogram* deep learning radiomic nomogram, *HL* Hosmer–Lemeshow

**Fig. 6:**
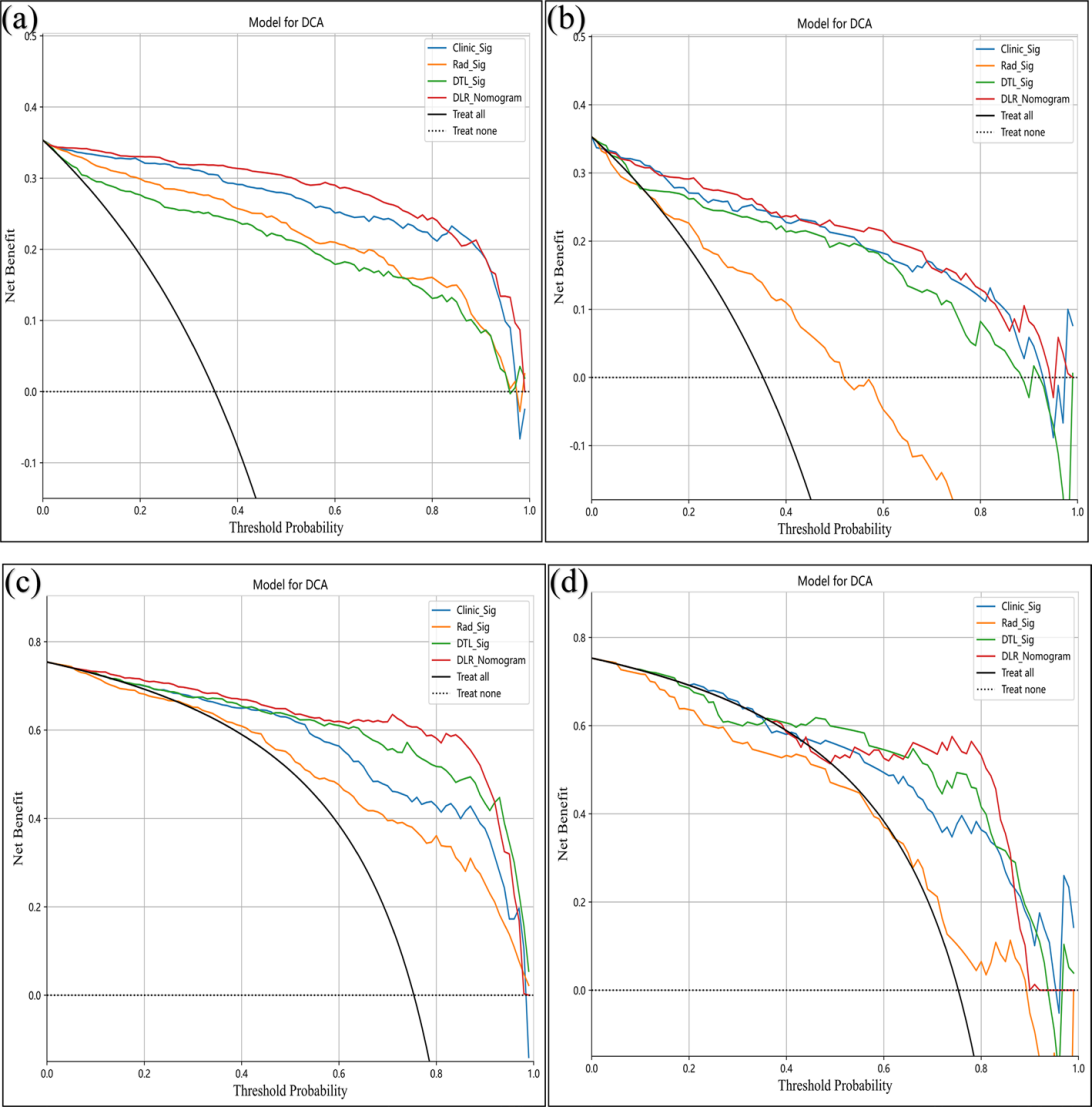
Decision curve analysis of the four models. **a** and **b**: Training and testing sets of task 1, respectively; **c** and **d**: training and testing sets of task 2, respectively. Note: Clinic_Sig, clinical signature; Rad_Sig, radiomics signature; DTL_Sig, deep transfer learning radiomic signature; DLR_Nomogram, deep learning radiomic nomogram

Decision curve analysis (DCA) results are shown in Fig. 6. In task 1, the DLR_Nomogram exhibited a greater net benefit, with risk threshold probabilities ranging from 0.15–0.80 for both the training and testing sets. In task 2, the DLR_Nomogram demonstrated a higher net benefit within the risk threshold probabilities between 0.10–0.90 for the training dataset and 0.65–0.85 for the testing dataset.

### Discussion

This study aimed to develop and validate a DLR_Nomogram, a non-invasive US-based tool, for the preoperative prediction of malignancy risk in 849 patients with ovarian tumours. The combined model incorporated Clinic_Sig, Rad_sig, and DTL_Sig and demonstrated superior diagnostic performance compared with one or two pre-fusion models. Furthermore, we conducted a comparative analysis between the predictive capabilities of the DLR_Nomogram and O-RADS. The DLR_Nomogram and O-RADS demonstrate notable diagnostic performance in forecasting ovarian malignancy risk in the testing set. Moreover, no statistically significant differences were observed between the two methods. Therefore, the predictive capabilities of the DLR_Nomogram and O-RADS in assessing the malignant propensity of ovarian tumours may be considered comparable.

Compared to alternative models, the DLR_Nomogram exhibited the most elevated AUC value in the training and testing sets, showcasing outstanding diagnostic competence and strong generalisability in predicting the malignant risk of O-RADS category 4 and 5 ovarian lesions.

Radiomics is an emerging tool in medical imaging that facilitates the extraction of high-dimensional imaging features. This tool captures more detailed tumour heterogeneity, surpassing the limitations of human visual perception and providing a more comprehensive depiction of tumour-related information [29]. Zhang et al. evaluated the diagnostic performance of magnetic resonance imaging-based radiomics in differentiating between benign and malignant adnexal tumours. The results of this study showed excellent accuracy of 0.90 in the leave-one-out cross-validation cohort and 0.87 in the independent validation cohort [30]. Similarly, Chiappa et al. examined the performance of a decision support system that utilised radiomics and machine learning to predict the risk of malignancy in ovarian tumours. This study revealed mean accuracies of 88% and 91% in the training and independent validation cohorts, respectively [31]. However, our investigations in tasks 1 and 2 revealed that handcrafted Rad_Sig did not exhibit notable benefits in discerning the malignancy risk of ovarian tumours.

DL has become increasingly common in image-pattern recognition [32]. A conventional CNN comprises numerous layers of neurons that can autonomously enhance its proficiency in identifying abstract image characteristics using a hierarchical analysis approach [21]. Given the constraints imposed by the limited size of medical datasets, a CNN pre-trained on ImageNet, referred to as TL, can be utilised to address related tasks effectively and mitigate the issue of overfitting resulting from inadequate training data [33]. TL has proven to be a valuable technique in current research since it leverages prior knowledge acquired from a similar classification task to enhance the performance of models trained on small samples [34].

Gao et al. developed a deep convolutional neural network (DCNN) model to automate the evaluation of US images and improve the diagnostic accuracy of ovarian cancer. The findings revealed that the DCNN model performed at a level comparable to US image experts and surpassed radiologists’ average diagnostic proficiency, thereby enhancing radiologists’ accuracy [35]. Similarly, Christiansen F. et al. reported that deep neural networks demonstrated accuracy comparable to that of US image experts in predicting ovarian malignancy [36]. Chen et al. devised a DL algorithm utilising multimodal US images and showed a diagnostic performance in distinguishing malignant from benign ovarian tumours that were on par with the subjective assessments of experts and the O-RADS system [37]. These findings indicate that CNN can play a significant role in accurately identifying different classifications of ovarian tumours. In this study, we used the ResNet50 and DenseNet121 models to assess the malignancy risk of ovarian tumours in tasks 1 and 2. In task 1, the AUC values for the training and testing sets were 0.915 and 0.890, respectively. In task 2, the AUC values for the training and testing sets were 0.937 and 0.853, respectively. These findings suggest that DTL_Sig has excellent predictive performance and robust generalisation capabilities.

The current area of interest in the field of tumour research involves the application of a model known as DLR, which combines manual radiomics with DTL. This model demonstrated its ability to improve the accuracy and reliability of predictions significantly. Numerous studies have consistently shown that DLR outperforms Rad_Sig and DTL_Sig when used individually [38–41]. The fusion of radiomics and DL in this model involved two distinct methods: feature and result fusion. However, feature fusion can often result in overfitting due to including more features [42]. Therefore, the fusion of the prediction results of Clinic_Sig, Rad_Sig, and DTL_Sig for the malignancy risk of individual ovarian tumours was performed in this study to create a new feature set. This new feature set was utilised as input for constructing a combined model using the logistic regression technique and subsequently visualised as a nomogram, referred to as the DLR_Nomogram. The DLR_Nomogram exhibited higher AUCs in the training and testing sets in tasks 1 and 2, and its predictive performance remained consistent with that in prior studies, surpassing that of one or two of the three individual models prior to fusion.

This study had several limitations. First, the study design was retrospective and conducted at a single centre, which may limit the generalisability of the findings. Additionally, the sample size was insufficient, warranting large-scale prospective multicenter studies to assess the applicability of the DLR_Nomogram in clinical practice comprehensively. Second, strict inclusion and exclusion criteria may have introduced a sample selection bias, potentially affecting the model’s training. Third, the extraction of features was limited to two-dimensional US images in single modes. However, including multimodal images such as colour Doppler images, spectral Doppler images, and CEUS could offer an incredible wealth of predictive information. Fourth, borderline ovarian tumour (BOT) was classified as malignant in this study owing to its malignant potential; however, it lacks interstitial invasion and exhibits a favourable prognosis. So, treatment and management strategies for BOT differ from those employed for malignant ovarian tumours. However, this study did not include further classification predictions. Fifth, the manually delineated region of interest (ROI) utilised in this study merely captured a single portion of the lesion, failing to account for the heterogeneity in the entire tumour. Last, the Grad-CAM solely elucidated the ROI of the deep neural networks without providing a comprehensive explanation of its underlying algorithmic principles. Consequently, the research foundations of DL should be explored further to expedite its integration into clinical practice.

### Conclusion

A DLR-Nomogram incorporating Rad_Sig, DTL_Sig, and Clinic_Sig was formulated and verified. The DLR_Nomogram exhibited a notable predictive accuracy comparable to that of the O-RADS and holds substantial potential in the preoperative assessment of the malignant propensity of ovarian tumours.

## Materials and methods

### Study population

This study included 849 patients diagnosed with ovarian tumours, confirmed by pathological examination, who underwent surgical resection at our hospital between July 2014 and October 2022.

The inclusion criteria were as follows: (1) completion of a US examination within one month before surgery and (2) availability of clear and definitive US images depicting the target lesion.

The exclusion criteria were as follows: (1) poor quality US images; (2) insufficient information in US images or clinical records; (3) simultaneous pregnancy; (4) presence of coexisting tumours in other locations or metastatic ovarian cancer; (5) prior treatment before US examination or surgery; and (6) uncertain or inconclusive pathological diagnosis results of the tissue obtained using a needle biopsy.

**Fig. 7:**
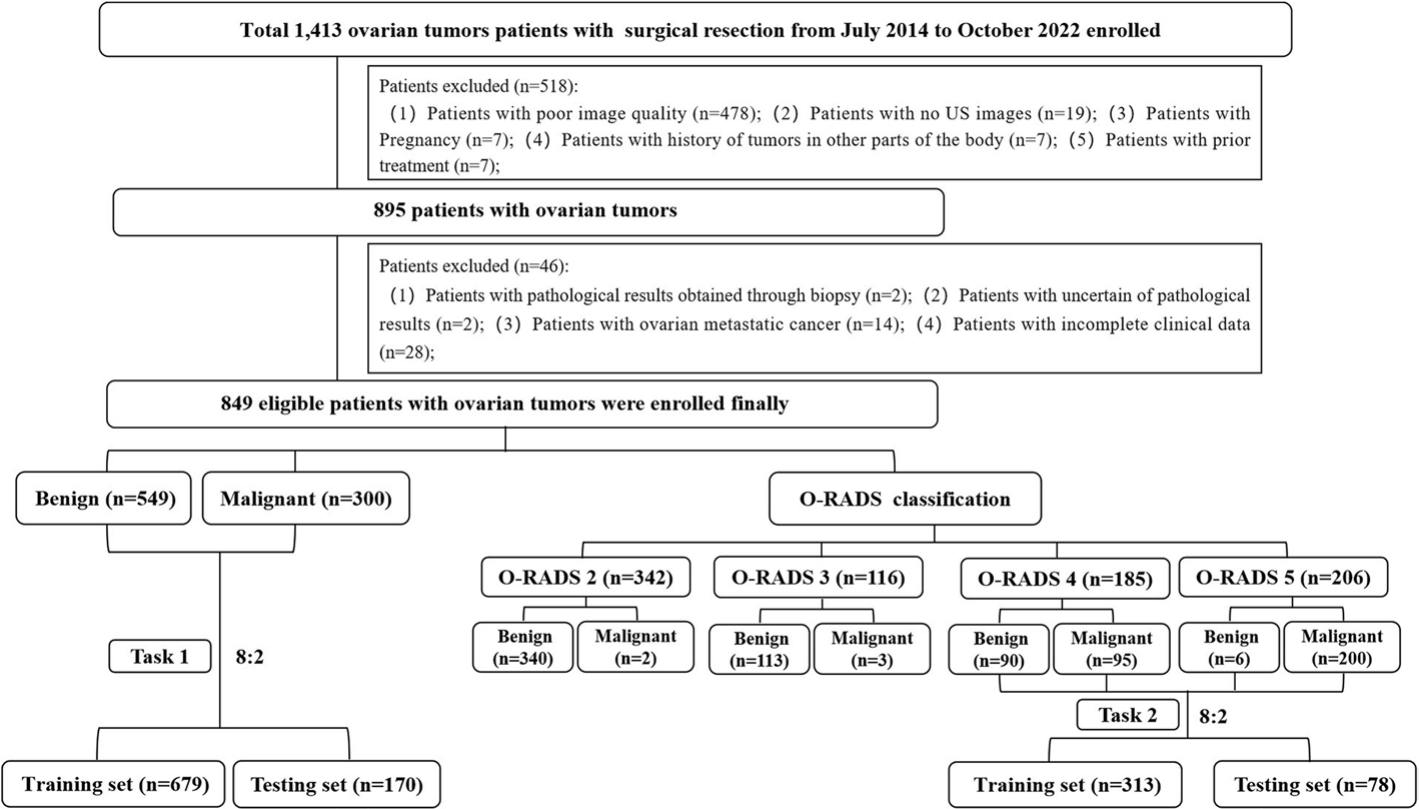
The flowchart depicting the process of this study. Note: US, ultrasound; O-RADS, ovarian-adnexal reporting and data system

The primary objective of this study was to investigate the DLR_Nomogram model in assessing the malignancy risk of ovarian tumours. We set up two tasks in this study. We predicted the malignancy risk of 849 patients with ovarian tumours and compared the predictive performance with that of O-RADS in task 1. Concurrently, we predicted the malignant risk of 391 ovarian lesions categorised as O-RADS 4 and 5 in task 2. The study process is illustrated in Fig. 7.

In this study, the reference standard for histopathological diagnosis was obtained using tumour resection surgery. The primary epithelial tumour, BOT, has a slow clinical course and lacks invasiveness. The cytological characteristics of BOT resemble those of malignant tumours, despite its non-invasive nature [18], leading to its malignant classification for statistical analysis in this study.

### Clinical data collection

The preoperative clinical characteristics of all patients were obtained from their electronic medical records, including age, menopausal status, height, weight, body mass index (BMI), CA125, red blood cell (RBC) count, white blood cell (WBC) count, neutrophil (N) count, lymphocyte (L) count, monocyte (M) count, platelet (PLT) count, and haemoglobin (Hb) levels. BMI and several inflammation-related risk factors, such as NLR, dNLR, PLR, LMR, and SII, were calculated using straightforward formulas:\begin{equation}\begin{aligned} & BMI=\frac{weight(kg)}{height^{2}(m^{2})}\\ & NLR={\frac{N(10^{9})}{L(10^{9})}}\\ & dNLR=\frac{N(10^{9})}{(WBC-N)(10^{9})}\\ & PLR=\frac{PLT(10^{9})}{L(10^{9})}\\ & LMR={\frac{L(10^{9})}{M(10^{9})}}\\ & SII=\frac{N(10^{9})\times PLT(10^{9})}{L(10^{9})}\end{aligned}\end{equation}

$${\text{BMI}} = \frac{{{\text{weight}}\left( {{\text{kg}}} \right)}}{{{\text{height}}^{2} \left( {{\text{m}}^{2} } \right)}},$$

$${\text{NLR}} = \frac{{N\left( {10^{9} } \right)}}{{L\left( {10^{9} } \right)}},$$

$${\text{dNLR}} = \frac{{N\left( {10^{9} } \right)}}{{\left( {{\text{WBC}} - N} \right)\left( {10^{9} } \right)}},$$

$${\text{PLR}} = \frac{{{\text{PLT}}\left( {10^{9} } \right)}}{{L\left( {10^{9} } \right)}},$$

$${\text{LMR}} = \frac{{L\left( {10^{9} } \right)}}{{M\left( {10^{9} } \right)}},$$

$${\text{SII}} = \frac{{N\left( {10^{9} } \right) \times {\text{PLT}}\left( {10^{9} } \right)}}{{L\left( {10^{9} } \right)}}.$$

### Ultrasound examination

All participants underwent transvaginal ultrasonography whenever feasible. Transabdominal ultrasonography was performed in cases where the tumour size prevented complete visualisation using transvaginal ultrasonography. Transrectal or transabdominal ultrasonography was performed if the patient was unsuitable for a transvaginal ultrasound examination. Some ultrasound equipment, such as GE Voluson E10, GE Voluson E8, GE Healthcare (GE Medical Systems, Zipf, Austria), and Mindray Resona R9, was used for data collection. The transducers used in this study included the RIC5-9-D and V11-3HU transvaginal probes and the C1-5-D and SC6-1U transabdominal probes. Various ultrasound semantic features were recorded, such as the maximum diameter of the lesion and its classification (≤ 50 mm, 50–100 mm, and ≥ 100 mm), the characteristics of the mass (cystic, mixed cystic and solid, and solid), the colour flow score (1, no flow signal; 2, small amount of blood flow signal; 3, moderate blood flow signal; and 4, enriched blood flow signals), the side of the lesion (unilateral or bilateral), and the presence or absence of ascites. The mass with the most complex morphological structure or the largest volume was selected when multiple ovarian-adnexal masses were present [18, 37, 43].

### O-RADS classification

The ACR formally published consensus guidelines for O-RADS US risk stratification and management in 2020. These guidelines classify the adnexal mass observed on US into six categories for risk classification: O-RADS 0, an incomplete evaluation; O-RADS 1, normal premenopausal ovary; O-RADS 2, almost undoubtedly benign lesion with < 1% risk of malignancy; O-RADS 3, low-risk lesion with 1–10% risk of malignancy; O-RADS 4, intermediate risk lesion with 10–50% risk of malignancy; and O-RADS 5, high-risk lesion with ≥ 50% risk of malignancy [17]. Ovarian-adnexal masses were categorised based on the O-RADS guidelines by Doctor A, a highly experienced gynaecology and obstetrics ultrasound specialist with a decade of professional practice. Doctor B, another gynaecology and obstetrics ultrasound expert with over 15 years of work experience, subsequently validated the classification. A senior gynaecology and obstetrics ultrasound expert with more than two decades of experience was consulted when the abovementioned doctors held differing opinions on establishing a consensus on categorisation. These doctors were unaware of the patient’s clinical and biochemical indicators or pathological results.

### Data preprocessing and delineation of the region of interest

Two-dimensional US images acquired from various US devices demonstrated notable dissimilarities in their grayscale ranges. We employed a sorting technique to arrange all grayscale values within each US image to counteract the biases arising from these disparities, subsequently limiting the range to the 0.5–99.5 percentile range for this investigation. Furthermore, the voxel spacing of US images obtained using different devices varied, necessitating the implementation of spatial standardisation techniques to alleviate the influence of such discrepancies. In this study, we employed a fixed-resolution resampling method to address the concerns above effectively.

Doctor A employed the ITK-SNAP 3.8.0 software (http://www.itksnap.org) to manually delineate the complete boundary of ovarian masses and identify the ROI. The ROI was validated by Doctor B. In cases of disagreement, consultation with a senior physician was sought to facilitate collaborative discussions and achieve consensus. Fifty patients were randomly selected from the dataset to ensure the reliability and replicability of the extracted radiomic features, and Doctor A re-delineated the ROIs after a two-week interval. Furthermore, Doctor C, a gynaecology and obstetrics ultrasound specialist with 12 years of work experience, autonomously delineated the ROIs. None of those mentioned above doctors had access to the patient’s clinical and biochemical parameters or pathological findings.

The maximum US image section depicting the ovarian lesion in each patient was chosen for this study to facilitate their input into the CNN. The grayscale values were normalised using the min–max transformation, resulting in a range of [−1, 1]. Subsequently, the resolution of each cropped subregion of the US image was adjusted to 224 × 224 using the nearest interpolation. The resulting US images were saved in the "PNG" format to adhere to the model’s input requirements.

### Extraction and selection of manual radiomics features

PyRadiomics is a software platform that is openly accessible and specifically designed to extract features from medical images. The process involved importing manually delineated ROI images into the PyRadiomics platform, where the features were extracted using an internal feature analysis program. Z-score normalisation was employed to address the issue of varying scales in manual radiomic features.

We assess the robustness and repeatability of radiomics features using the ICC. Features with an ICC ≥ 0.85 were deemed highly robust and suitable for further analysis. All radiomic features underwent T-tests or Mann–Whitney U tests, and only the features with a *P*-value < 0.05 were retained. Spearman correlation analysis was conducted to determine the correlation coefficients of features exhibiting high repeatability. If the correlation coefficient between the two features exceeded 0.9, then either feature was retained [44]. Feature filtering was performed using a greedy recursive deletion strategy, eliminating the features with the highest redundancy in the current dataset.

The training set underwent feature selection using LASSO regression from the scikit-learn package in Python. LASSO regression effectively reduces the regression coefficients toward zero and accurately assigns zero coefficients to irrelevant features, depending on the regularisation weight *λ*. A tenfold cross-validation was performed to determine the optimal *λ*, and the final value of *λ* was chosen based on the minimal cross-validation error. Features with nonzero coefficients were retained for subsequent development of the Rad_Sig.

### The deep transfer learning radiomics procedure

In this study, the pre-trained CNN model, TL, trained on the ImageNet dataset, was employed to mitigate the overfitting problem arising from the training dataset’s constrained size. The presence of imbalanced or inadequate data when applying DTL for image classification frequently requires the implementation of data augmentation. In the study, we employed horizontal flipping and random cropping to augment the sample size, enhance the model’s accuracy, and improve its generalisation capacity.

### Signature building

Following feature selection using LASSO regression, the resultant features were utilised as inputs for the logistic regression model to establish manual Rad_Sig.

The US image encompassing the largest section of the ovarian tumour was fed into the CNN. Following an intricate internal algorithm, the resulting prediction probability for each sample was referred to as the DTL_Sig. Grad-CAM was used to visualise the hidden layer network algorithm and elucidate the decision-making process of the CNN model.

Statistical analysis was conducted on the baseline clinical data. Clinical parameters and US semantic features with a *P* value < 0.05 were chosen. Spearman correlation analysis was employed to ascertain the linear association among these parameters, whereas parameters lacking significant linear relationships were input into the logistic regression model to construct the Clinic_Sig.

We integrated the predictive results of Rad_Sig, DTL_Sig, and Clinic_Sig for each sample to create a new feature set to intuitively and efficiently assess the incremental prognostic value of Rad_Sig and DTL_Sig for clinical risk factors. The new feature set was input into the logistic regression model to construct a combined model based on the training set, which was visualised as a nomogram, namely, the DLR_Nomogram.

### Model evaluation

The models’ performances were evaluated using ROC and the AUC. Additionally, quantitative measures such as accuracy, precision, recall, and F1 scores were employed. The DeLong test was used to compare the models’ diagnostic performance differences [45]. Model fit was assessed using the HL test [46]. A calibration curve was constructed to visualise the results of the HL test. DCA [47] was used to quantitatively evaluate the overall benefit of the predictive model across various threshold probabilities.

**Fig. 8:**
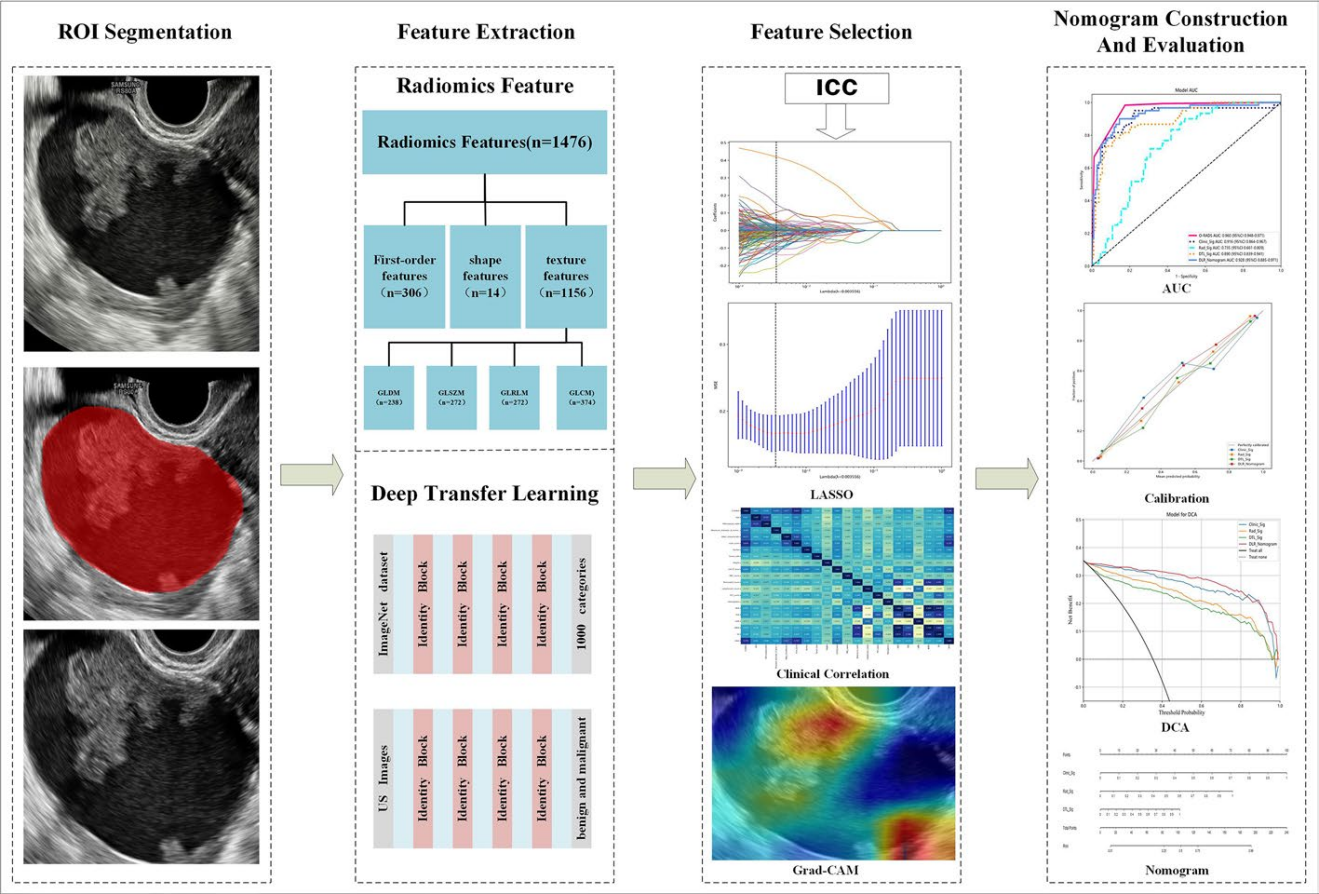
The workflow of US-based DLR_nomogram. Note: DLR, deep learning radiomics; US, ultrasound; ROI, region of interest; ICC, intraclass correlation coefficient; LASSO, least absolute shrinkage and selection operator; Grad-CAM, gradient-weighted class activation mapping; AUC, area under the curve; DCA, decision curve analysis; GLDM, gray level dependence matrix; GLSZM, gray level size zone matrix; GLRLM, gray level run length matrix; GLCM, gray level co-occurrence matrix

The procedure for constructing the DLR-Nomogram is depicted in Fig. 8.

### Statistical methods

We employed statistical analysis to compare differences in continuous variables, such as t-tests or Mann–Whitney U tests, whereas Chi-squared or Fisher’s exact tests were used for categorical variables. Statistical analyses and data visualisation were performed using SPSS (version 27.0; IBM Corp.) and Python (https://www.python.org/). Two-tailed tests with a *P* value of < 0.05 were considered statistically significant.

The testing set and machine learning algorithm employed were held constant to conduct an impartial and equitable comparison of the disparities between the models, and the hyperparameters utilised in the model training procedure were kept as consistent as possible.

### Supplementary Information

**Additional file 1: Table S1.** The baseline characteristics of task 1. **Table S2.** The baseline characteristics of task 2 [30].

**Additional file 2: Figure S1.** The ROC curve of the O-RADS classification. Note: O-RADS, Ovarian-adnexal reporting and data system; AUC, Area under the curve; CI: Confidence interval; Ac: Accuracy; Se: Sensitivity; Sp: Specificity.

**Additional file 3: Figure S2.** Spearman correlation analysis. **a** task 1; **b** task 2. Note: O-RADS, Ovarian-adnexal reporting and data system; RBC, Red blood cell; PLT, Platelet; NLR, Neutrophil-to-lymphocyte ratios; PLR, Platelet-to-lymphocyte ratios; LMR, Lymphocyte-to-monocyte ratios; dNLR, derived Neutrophil-to-lymphocyte ratios; SII, Systemic immune-inflammation index; CA125, Carbohydrate antigen 125.

**Additional file 4: Figure S3.** The ratio of handcrafted radiomics features of tasks 1 and 2. **Note:** GLDM, Gray level dependence matrix; GLSZM, Gray level size zone matrix; GLRLM, Gray level run length matrix; GLCM, Gray level co-occurrence matrix.

**Additional file 5: Figure S4.** All handcrafted radiomics features’ corresponding *P*-value results. **a** task 1; **b** task 2. Note: GLDM, Gray level dependence matrix; GLSZM, Gray level size zone matrix; GLRLM, Gray level run length matrix; GLCM, Gray level co-occurrence matrix

**Additional file 6: Figure S5.** The selected handcrafted radiomics features and corresponding weight. **a** task 1; **b** task 2.

## Data Availability

The datasets used and analysed during the current study are available from the corresponding author on reasonable request.

## Abbreviations

CA125: Carbohydrate antigen 125
PLR: Platelet-to-lymphocyte ratio
NLR: Neutrophil-to-lymphocyte ratio
dNLR: Derived Neutrophil-to-lymphocyte ratio
LMR: Lymphocyte-to-monocyte ratio
SII: Systemic immune-inflammation index
CAR: C-reactive protein–albumin ratio
US: Ultrasound
ACR: American College of Radiology
O-RADS: Ovarian-adnexal reporting and data system
AUC: Area under the ROC curve
DL: Deep learning
CNN: Convolutional neural network
TL: Transfer learning
DTL: Deep transfer learning
DLR_Nomogram: Deep learning radiomics nomogram
Clinic_Sig: Clinical signature
GLDM: Gray-level dependence matrix
GLSZM: Gray-level size zone matrix
GLRLM: Gray-level run length matrix
GLCM: Gray-level co-occurrence matrix
ICC: Intra-/inter-class correlation coefficient
LASSO: Least absolute shrinkage and selection operator
Rad_Sig: Radiomics signature
DTL_Sig: Deep learning radiomic signature
Grad-CAM: Gradient-weighted class activation mapping
CEUS: Contrast-enhanced ultrasound
ROC: Receiver operating characteristic curve
CI: Confidence interval
HL: Hosmer–Lemeshow
DCA: Decision curve analysis
DCNN: Deep convolutional neural network
BOT: Borderline ovarian tumour
ROI: Region of interest
BMI: Body mass index
RBC: Red blood cell
WBC: White blood cell
N: Neutrophil
L: Lymphocyte
M: Monocyte
PLT: Platelet
Hb: Haemoglobin
