# Supplementary figures and images for: Development and validation of an ultrasound-based deep learning radiomics nomogram for predicting the malignant risk of ovarian tumours
Source: Biomed Eng Online. 2024 Apr 9;23:41. doi: 10.1186/s12938-024-01234-y (PMC11003110; doi:10.1186/s12938-024-01234-y)

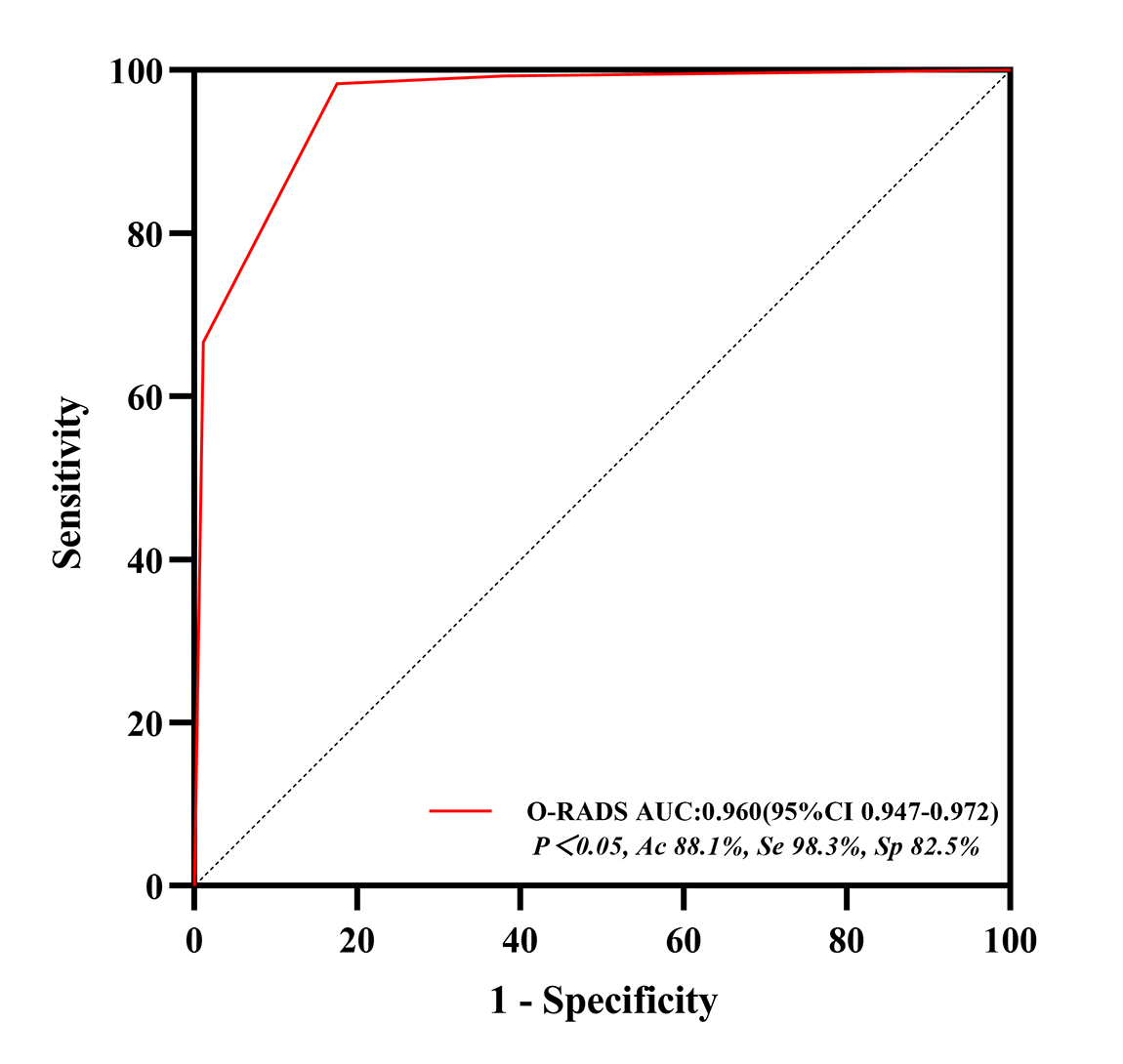

Supplement: Supplementary file 2 — Additional file 2: Figure S1. The ROC curve of the O-RADS classification. Note: O-RADS, Ovarian-adnexal reporting and data system; AUC, Area under the curve; CI: Confidence interval; Ac: Accuracy; Se: Sensitivity; Sp: Specificity. [file 12938_2024_1234_MOESM2_ESM.tif]

## Slide 1
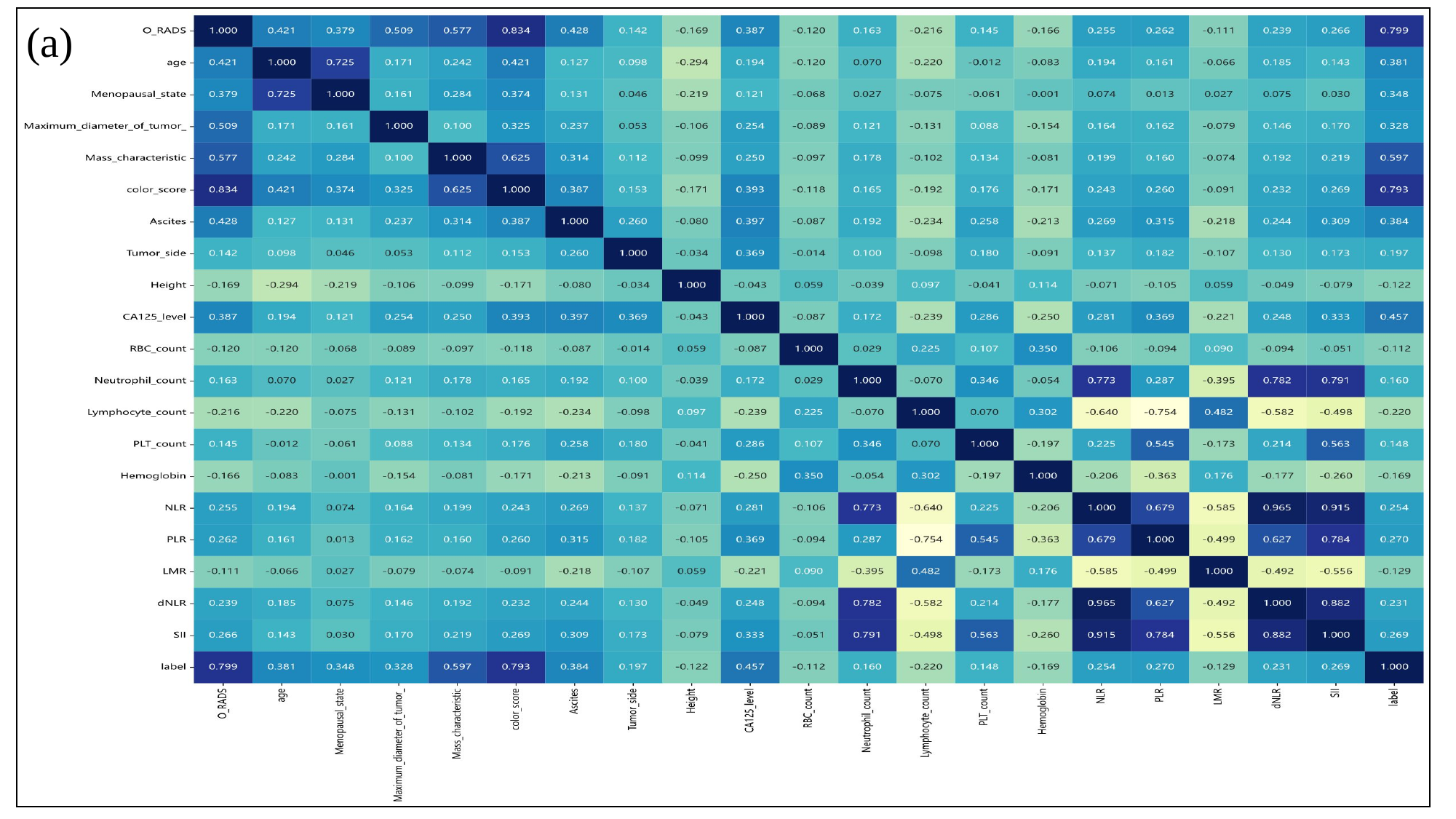

(a)

## Slide 2
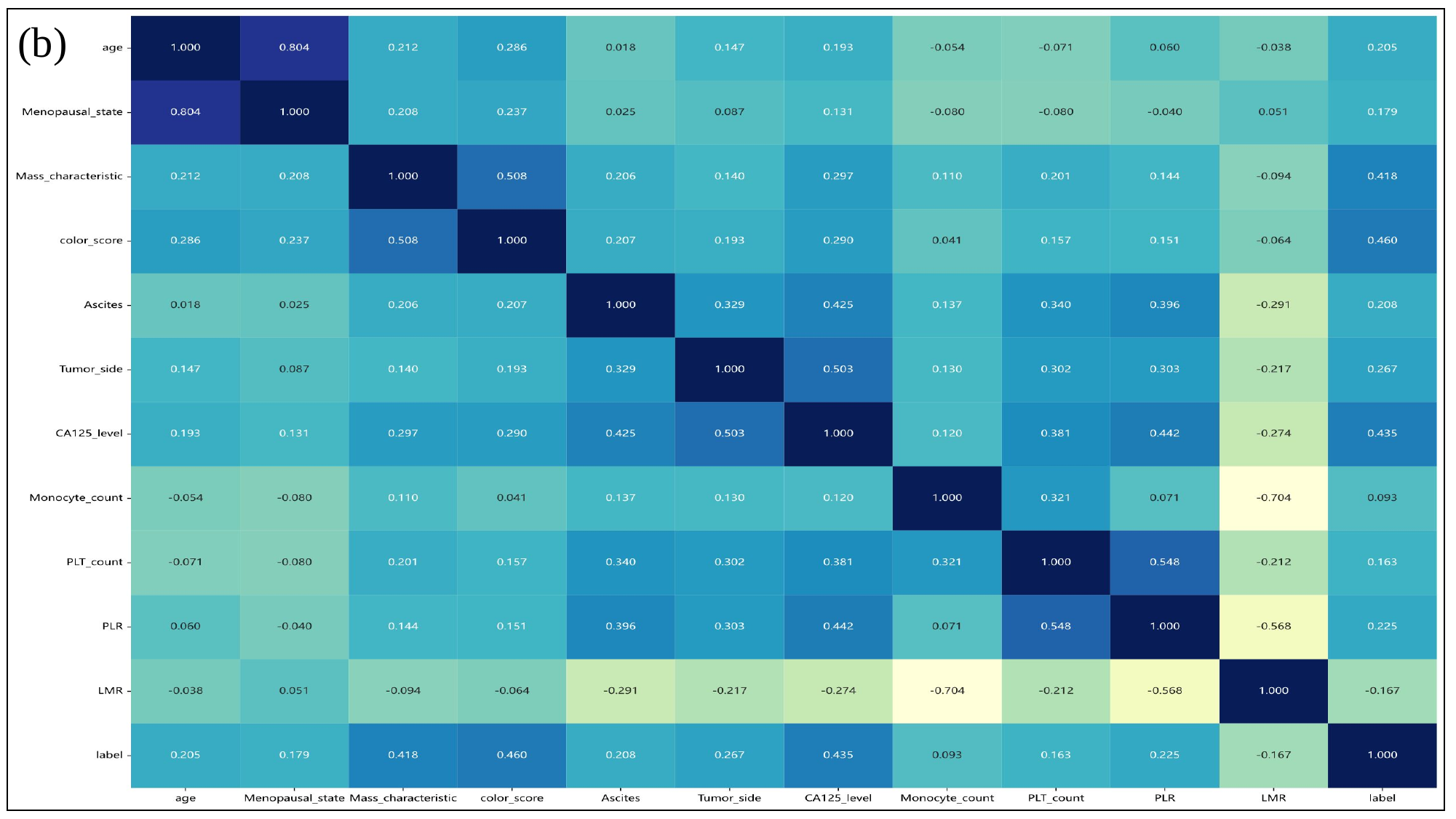

(b)

Supplement: Supplementary file 3 — Additional file 3: Figure S2. Spearman correlation analysis. a task 1; b task 2. Note: O-RADS, Ovarian-adnexal reporting and data system; RBC, Red blood cell; PLT, Platelet; NLR, Neutrophil-to-lymphocyte ratios; PLR, Platelet-to-lymphocyte ratios; LMR, Lymphocyte-to-monocyte ratios; dNLR, derived Neutrophil-to-lymphocyte ratios; SII, Systemic immune-inflammation index; CA125, Carbohydrate antigen 125. [file 12938_2024_1234_MOESM3_ESM.pptx]

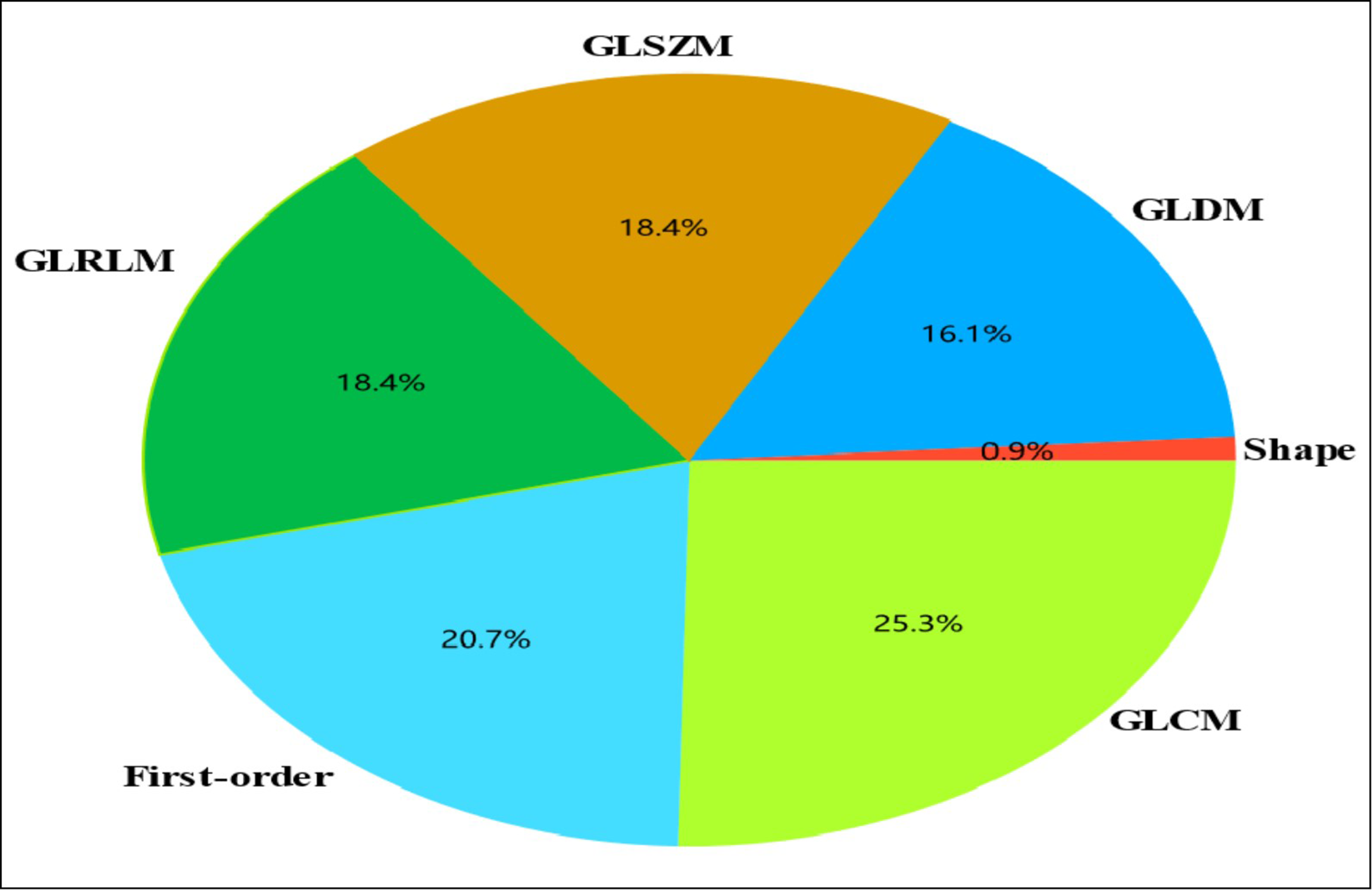

Supplement: Supplementary file 4 — Additional file 4: Figure S3. The ratio of handcrafted radiomics features of tasks 1 and 2. Note: GLDM, Gray level dependence matrix; GLSZM, Gray level size zone matrix; GLRLM, Gray level run length matrix; GLCM, Gray level co-occurrence matrix. [file 12938_2024_1234_MOESM4_ESM.tif]

## Slide 1
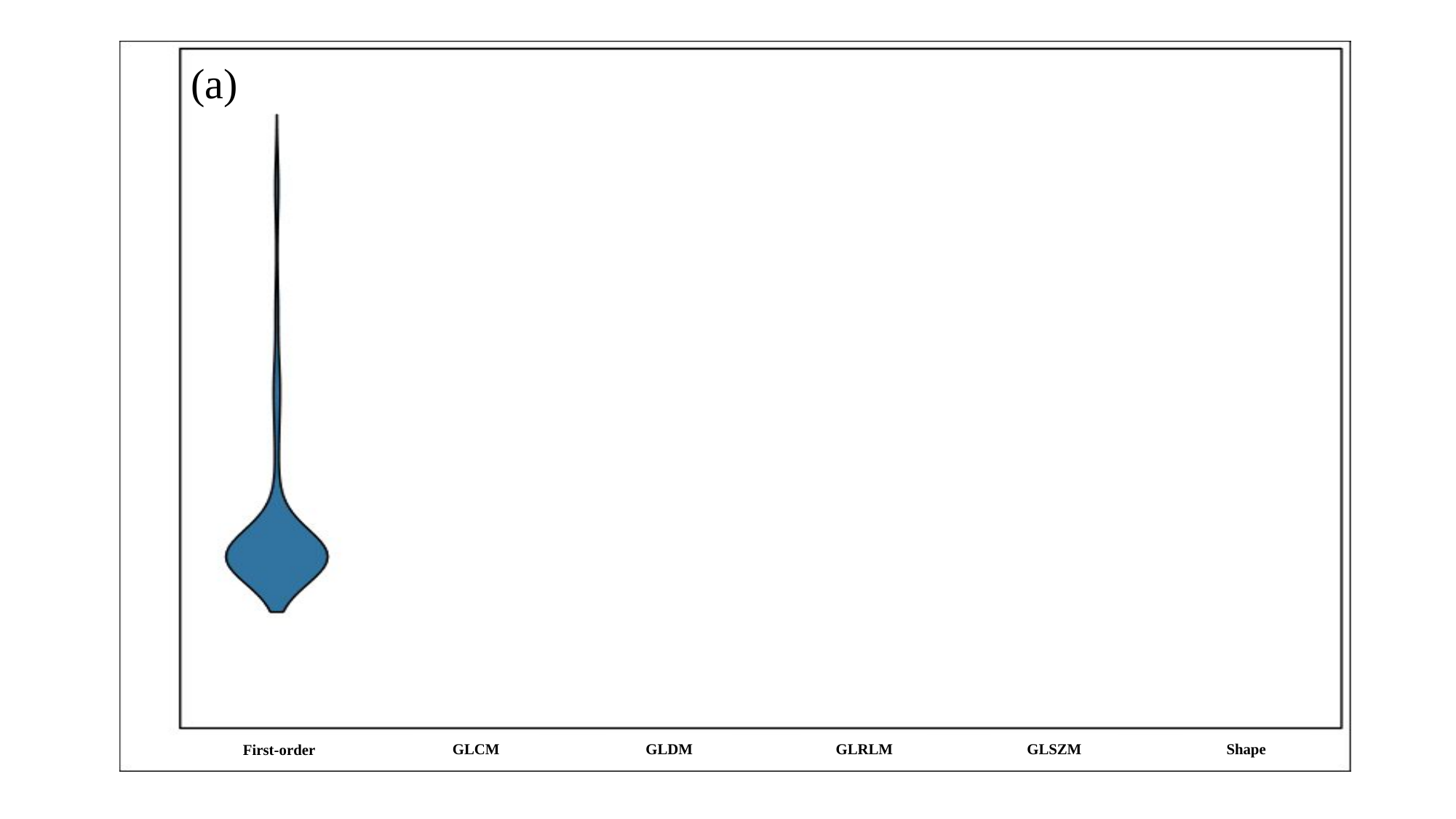

GLDM
GLSZM
Shape
GLCM
GLRLM
First-order
(a)

## Slide 2
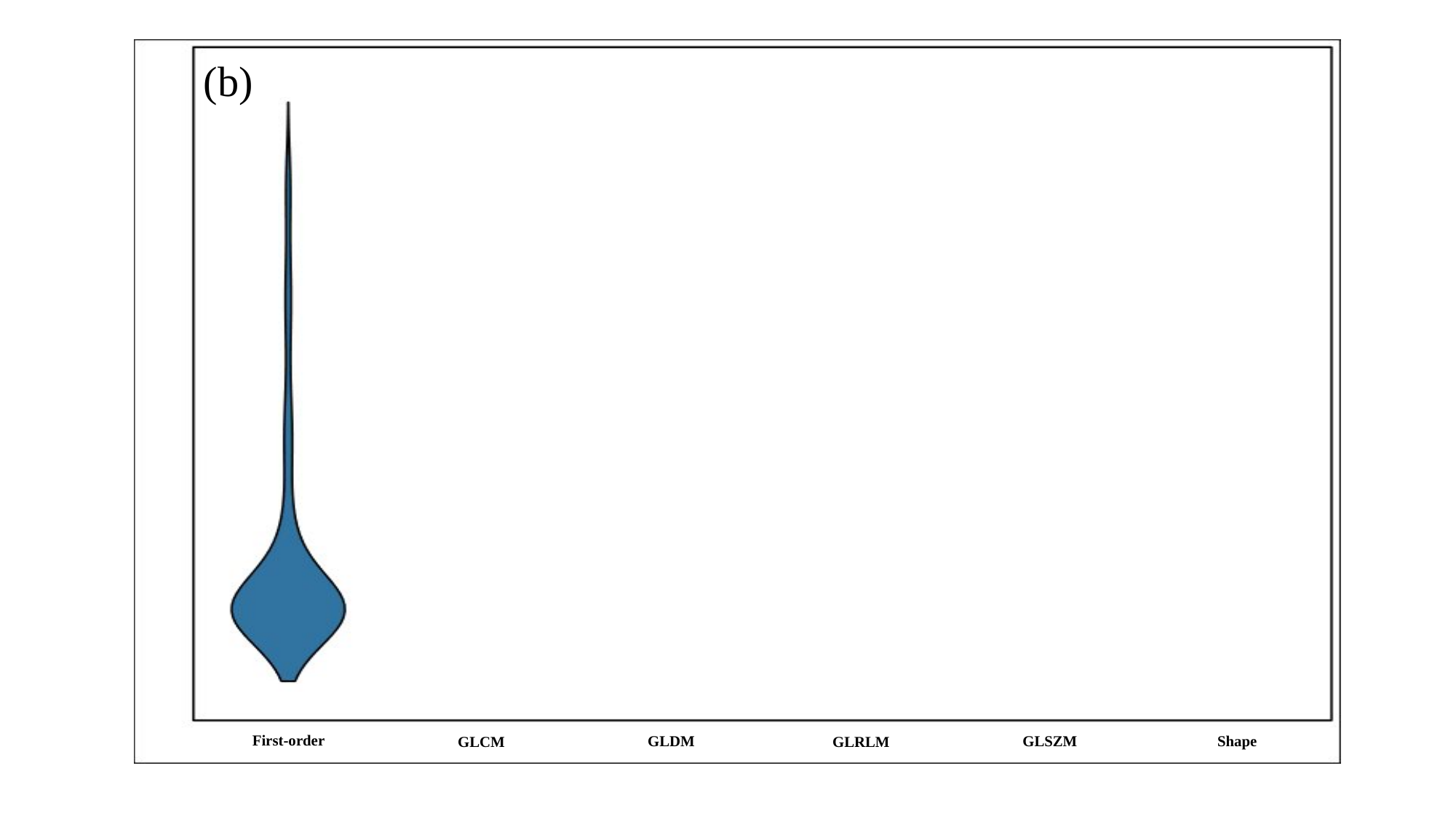

First-order
GLDM
GLSZM
Shape
GLCM
GLRLM
(b)

Supplement: Supplementary file 5 — Additional file 5: Figure S4. All handcrafted radiomics features’ corresponding P-value results. a task 1; b task 2. Note: GLDM, Gray level dependence matrix; GLSZM, Gray level size zone matrix; GLRLM, Gray level run length matrix; GLCM, Gray level co-occurrence matrix [file 12938_2024_1234_MOESM5_ESM.pptx]

## Slide 1
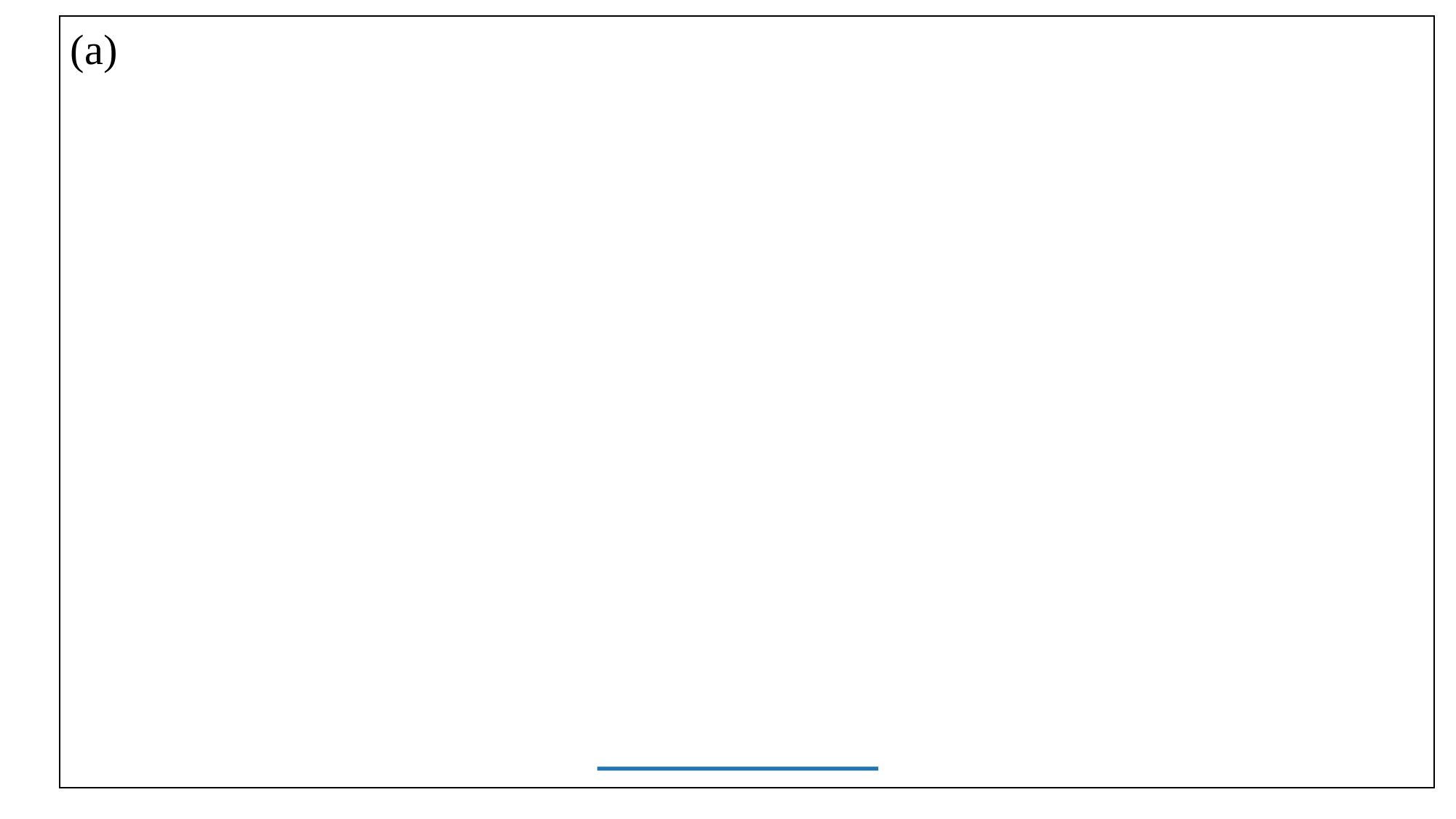

(a)

## Slide 2
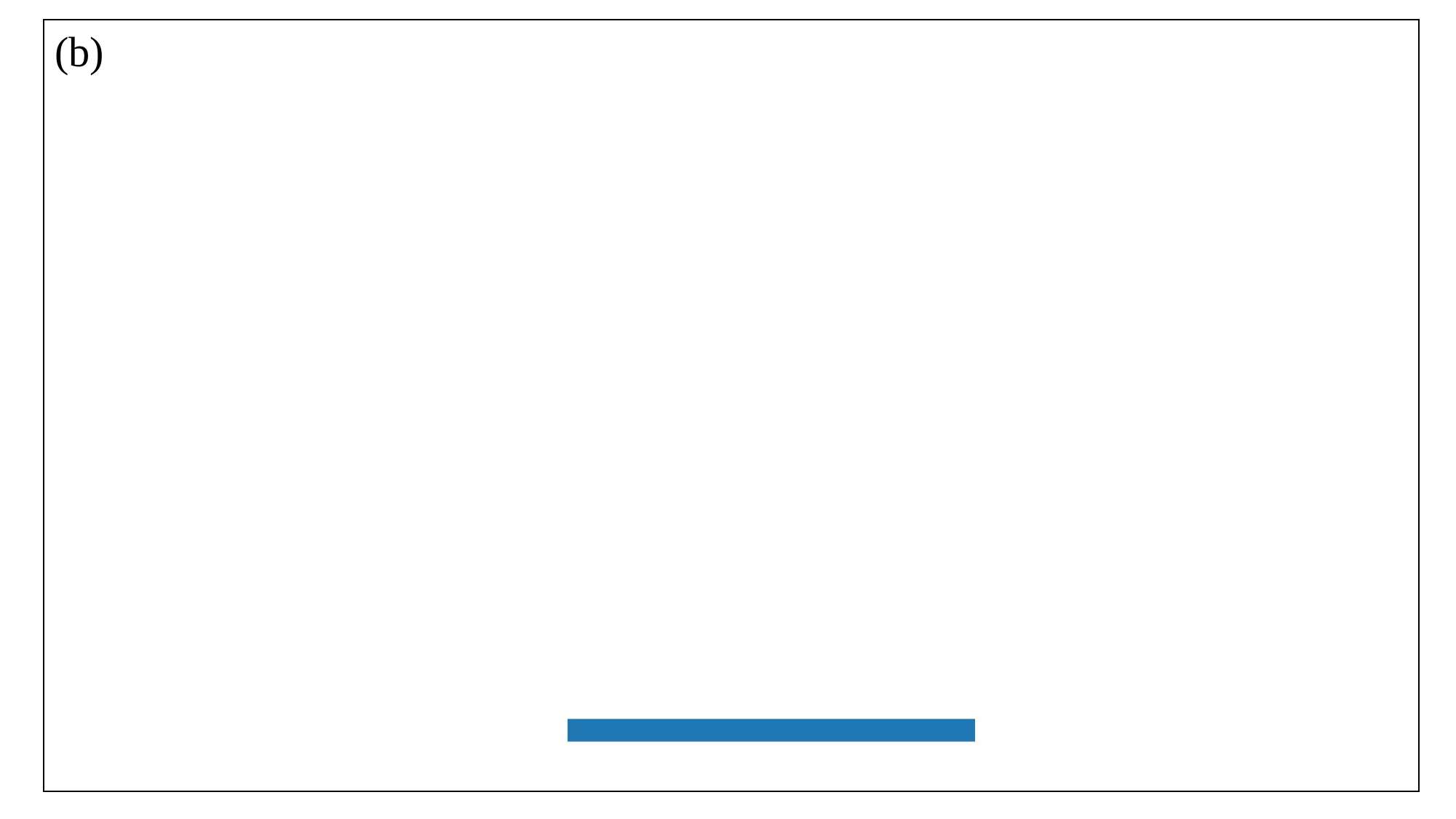

(b)

Supplement: Supplementary file 6 — Additional file 6: Figure S5. The selected handcrafted radiomics features and corresponding weight. a task 1; b task 2. [file 12938_2024_1234_MOESM6_ESM.pptx]
